# Phytochemical Characteristics and Anti-Inflammatory, Immunoregulatory, and Antioxidant Effects of *Portulaca oleracea* L.: A Comprehensive Review

**DOI:** 10.1155/2023/2075444

**Published:** 2023-08-31

**Authors:** Vahideh Ghorani, Saeideh Saadat, Mohammad Reza Khazdair, Zahra Gholamnezhad, Hesham El-Seedi, Mohammad Hossein Boskabady

**Affiliations:** ^1^Clinical Research Development Unit, Imam Reza Hospital, Faculty of Medicine, Mashhad University of Medical Sciences, Mashhad, Iran; ^2^Applied Biomedical Research Center, Mashhad University of Medical Sciences, Mashhad, Iran; ^3^Department of Physiology, School of Medicine, Zahedan University of Medical Sciences, Zahedan, Iran; ^4^Cardiovascular Diseases Research Center, Birjand University of Medical Sciences, Birjand, Iran; ^5^Department of Physiology, Faculty of Medicine, Mashhad University of Medical Sciences, Mashhad, Iran; ^6^International Research Center for Food Nutrition and Safety, Jiangsu University, Zhenjiang 212013, China; ^7^International Joint Research Laboratory of Intelligent Agriculture and Agri-Products Processing, Jiangsu University, Zhenjiang 210024, China; ^8^Department of Chemistry, Faculty of Science, Menoufia University, Shebin El-Kom 31100107, Egypt

## Abstract

*Portulaca oleracea* L. (*P. oleracea*) or purslane is a plant from the *Portulacaceae* family, which is used as food and traditional medicine for various diseases. This review article provides comprehensive information on the antioxidant, immunomodulatory, and anti-inflammatory properties of *P. oleracea* and its constituents. The literature survey of the different databases until the end of June 2023 was explored based on the keywords including the “*P. oleracea*, purslane, anti-inflammatory, immunomodulatory, and antioxidant properties.” The plant contains flavonoids, alkaloids, terpenoids, fatty acids, vitamins, minerals, and some other compounds. The results indicated that *P. oleracea* and its constituents showed anti-inflammatory and immunomodulatory properties through reduction of inflammatory mediators including interferon gama (IFN-*γ*), interleukin (IL)-10, IL-4, tumor necrosis factor-alpha (TNF-*α*), and nitric oxide. Improvement in cytokines' serum levels (IFN-*γ*, IL-10, and IL-4) and increased IgG and IgM serum levels, as well as reduction of IgE, phospholipase A2, and total protein were demonstrated for *P. oleracea*. The plant and its constituents also improved oxidative stress by reduction of oxidant and increase of antioxidant markers. *P. oleracea* could be considered as an effective remedy for various inflammatory and immune diseases.

## 1. Introduction

*Portulaca oleracea* L. (*P. oleracea*) is a plant with an annual life cycle from the *Portulacaceae* family. The *Portulacaceae* includes 25–30 genera making it the largest genus Portulaca [1]. It has a global geographical distribution specifically in the tropical and subtropical areas including South America, Australia, and Africa [2, 3]. This plant is known as purslane and has thick fleshy leaves, yellow flowers, and small black seeds, and its stem may reach 15.30 cm in height [4].

Different sections of the plant such as stems, leaves, and flower buds are edible [5, 6]; thus, it is used both as a plant food and medicinal herb in the eastern Mediterranean and middle eastern from Italy to China [4]. In traditional medicine, *P. oleracea* was advised for the therapy of several conditions including gastrointestinal and respiratory disorders, inflammations, kidney and liver diseases, and headaches [4, 7–10]. In addition, the various pharmacological properties of *P. oleracea* were investigated and proved effective in analgesia and inflammation [11], antioxidation [12], and nephroprotection [13] and as antitussive [14], antimicrobial, anticancer, antidiabetic, antiulcerogenic, neuroprotective, hepatoprotective in wound healing and hypocholesterolemic activities [4, 5, 15, 16], as well as the relaxant effect due to inhibition of muscarinic receptors [12], stimulation of *β*-adrenoceptors in isolated guinea pigs' tracheal smooth muscles [17], and bronchodilatory effect in asthmatic patients [1, 13, 18]. Hence, pharmaceutical properties of *P. oleracea* were mentioned in most prominent medical textbooks including Canon of Medicine by Avicenna, Al-Hawi by Rhazes Zakhireh Kharazmshahi by Jorjani, and other Traditional Persian Medicine (TPM) books [4]. *P. oleracea* is also indexed in a number of pharmacopoeias such as the Ayurvedic Pharmacopoeia of India [19] and Pharmacopoeia of PR China [20].

Pharmacokinetics of quercetin, a constituent of *P. oleracea*, showed that after oral administration of a single dose (10 mg/kg) of quercetin in rats, about 6.7% was absorbed in the form of unchanged quercetin [21]. However, high amounts of conjugated quercetin are found in the systemic circulation. 93.3% of quercetin was metabolized in the gut and only 3.1% metabolized in the liver [21]. Pharmacokinetic of quercetin in humans was also indicated that after the oral administration of quercetin (500 mg three times daily); the clearance (CL/F) was high (3.5 × 10^4^ l/h) with an average terminal half-life of 3.5 h for quercetin. The maximum concentration (*C*_max_) value for the quercetin-conjugated metabolites was 447.8 ng/ml [22].

Oral absorption and disposition of alpha-linolenic acid (another constituent of *P. oleracea*) after administration of a single dose of 3000 mg enriched goat dairy fat (EDF) containing 31 mg ALA/kg body weight to rats were evaluated. ALA was rapidly absorbed (*t*_1/2a_, 76 h) and slowly eliminated (t1/2*β*, 16.52 h), for plasma. *C*_max_ value in plasma was 63.42 *μ*g/mL [23].

Anti-inflammatory, antioxidant, immunomodulatory, and antitumor activities of *P. oleracea* were briefly described in a review paper [1]. However, this article is an updated and comprehensive narrative review of the pharmacological properties of *P. oleracea* based on evidence from animal and human studies. All attempts have been made to make a very detailed literature, surveying and as comprehensive as possible. Assessment of the quality of the individual studies is not included in this review, and it will be performed under quantitative systematic reviews (i.e., meta-analysis) in future studies.

## 2. Methods

In this review article, the different databases such as ScienceDirect, PubMed, and Scopus were searched until the end of June 2023 to identify studies published regarding the anti-inflammatory, immunoregulatory, and antioxidant effects of *P. oleracea* and its constituents. The keywords used for the search were *Portulaca* oleracea, purslane, *P. oleracea* constituents, anti-inflammatory, immunomodulatory, and antioxidant. The studies obtained from the abovementioned databases were screened by two authors separately, the search results were checked and finally articles, chapters, and thesis copies related to the topic of this review that were available online were included. The included articles were reviewed by authors, and information from each study was presented. Articles in a language other than English, abstracts, or unpublished articles were excluded.

## 3. Phytochemistry

**Figure 1:**
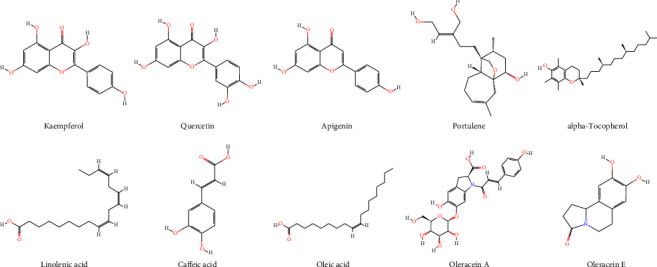
The main active constituents of *P. oleracea*.

Several constituents have been isolated from *P. oleracea* including flavonoids, alkaloids, terpenoids, carotenoids, fatty acids, sterols, polysaccharides, proteins, vitamins, and minerals [4, 10]. Compounds isolated from *P. oleracea* are presented in Table 1. The main active constituents of the plant are also shown in Figure 1.

### 3.1. Flavonoids

The amount of flavonoids changes in the various sections of the plant. They are the highest amounts in the roots and subsequently in the stems and leaves; apigenin, genistein, genistin, kaempferol, luteolin, myricetin, and quercetin are seven flavonoids in this plant. However, in ethanolic extracts of leaves and stems, only apigenin and kaempferol have been found with the levels in the former being higher [10, 25, 26]. Portulacanones A–D are homoisofalvonoids with the same chemical structure, which have been derived from aerial parts of the plant [4, 24].

### 3.2. Alkaloids

Alkaloids such as dopa, dopamine, noradrenalin, and N-trans-feruloyltyramine are other components of this plant. The dopamine and noradrenalin are found in greater amounts in leaves relative to stem and seeds. The amount of two alkaloids isolated from leaves varies based on solvents used in the extraction process [30, 31]. Oleraceins A–E, cyclodopa alkaloids, 1,5-dimethyl-6-phenyl-1,2-dihydro-1,2,4-triazin-3(2 H)-one, and (3 R)-3,5-bis(3-methoxy-4-hydroxyphenyl)-2,3-dihydro-2(1 H)-pyridinone are other alkaloids, derived from this plant [10, 27, 47]. Oleracone was the first isolated alkaloid from *P. oleracea* and showed significantly anti-inflammatory properties in macrophages stimulated by lipopolysaccharides [4].

### 3.3. Terpenoids

Portuloside A and B, monoterpene glycosides, and portulene, which is a diterpene, have been derived from *P. oleracea*. In addition, *P. oleracea* contains triterpenes [4, 32–34].

### 3.4. Fatty Acids

*P. oleracea* has been shown to be one of main sources of omega-3 fatty acids, in particular, *α*-linolenic acid, and other essential fatty acids such as linoleic, oleic, palmitic, palmitoleic, docosahexaenoic, and stearic eicosapentaenoic acids [4, 37–39].

### 3.5. Vitamins and Minerals

*P. oleracea* is rich in vitamins such as vitamin A, B-complex vitamins (thiamin, riboflavin, niacin, pantothenic acid, pyridoxine, and folates), ascorbic acid, and *α*-tocopherol. Minerals such as calcium, potassium, magnesium, manganese, phosphorus, copper, selenium, zinc, and iron have also been derived from this plant [4, 39, 44].

### 3.6. Other Compounds

Portulacerebroside A, catechol, bergapten, glutathione, beta-sitosterol, daucosterol, proline, chlorophyll, tannin, isopimpinellin, pectins, and melatonin have also been isolated from *P. oleracea* [4, 10].

## 4. Anti-Inflammatory Properties

Inflammation is a defensive reaction of the tissue to injury, which in the acute stage is characterized by accumulation of inflammatory cells (leukocytes) and mediators as well as increase in the vascular permeability. Different cytokines such as TNF-*α*, interleukin (IL)-1 (*α* and *β*), IL-6, IL-8, and IL-11 play effective roles in acute inflammatory responses [48]. Inflammatory mediators are metabolites of arachidonic acid, chemokines, cytokines, and free radicals which lead to enhanced cell proliferation, angiogenesis, mutagenesis, and oncogene activation [49]. Although nonsteroidal and steroidal anti-inflammatory agents are currently described for the improvement of acute and chronic inflammatory conditions like rheumatoid arthritis (RA), however, they showed considerable side effects in long-term medication [50–52]. Therefore, new and safe anti-inflammatory agents are needed, and medical herbs and their active ingredients are one of the best candidates for new drugs and effective remedies [53]. Plants and their active components are potential candidates for ongoing research for this purpose.

### 4.1. Anti-Inflammatory Properties of *P. oleracea*

#### 4.1.1. *In Vitro* Studies

An investigation by Askari et al. showed that incubation of human lymphocytes with the hydroethanolic extract of *P. oleracea* (10, 40, and 160 *µ*g/ml) in nonstimulated and stimulated conditions significantly increased IL-10, while decreasing IL-4 [54]. In nonstimulated cells, the plant extract also significantly decreased cell proliferation compared to the nontreated group. In addition, IL-4, IL-10, interferon gama (IFN-*γ*), and nitric oxide (NO) production significantly decreased in stimulated lymphocytes following treating with the plant extract compared to nontreated cells [54]. In a study by Lee et al., pretreatment of human umbilical vein endothelial cells by various doses of *P. oleracea* aqueous extract inhibited TNF-*α* induced intracellular reactive oxygen species (ROS) production [55]. In addition, the extracts of the plant suppressed TNF-*α*-induced overexpression of E-selectin and adhesion molecules dose dependently. Moreover, *P. oleracea* extract significantly inhibited TNF-*α*-induced degradation of I*κ*B-*α* (a member of a family of cellular proteins) on TNF-*α*-induced nuclear factor-kappa B (NF-*κ*B) binding in the vascular endothelial cells [55]. Productions of NO, TNF-*α*, IL-1*β*, and IL-6 were inhibited by *P. oleracea* ethanol extract in RAW 264.7 cells (a cell line derived from mice) induced by LPS [56]. In addition, the phosphorylation of ERK1/2, c-Jun NH_2_-terminal kinase (JNK), and NF-*κ*B activation in the cells were suppressed by the extract of the plant [56]. In another study, treatment with 250 *µ*g/ml of the polysaccharide fraction of *P. oleracea* (POL-P3b) led to overexpression of cluster of differentiation-80 (CD80), CD83, and CD86. Productions of IL-12, TNF-*α*, and to a lesser extent IL-10 were also upregulated by POL-P3b [57]. The increase of the toll-like receptor 4 (TLR-4) expression is caused by treatment with POL-P3b on dendritic cells (DCs). Therefore, DCs maturation may occur by POL-P3b via TLR-4 [57].

In a study conducted by Kim et al., ethanolic extract of *P. oleracea* reduced NO production and suppressed the mRNA expression of the inflammatory parameters such as TNF-*α* and IL-1-*β* on LPS-induced inflammation in RAW 264.7 cells [58]. In addition, incubation of LPS-stimulated human peripheral blood mononuclear cells (PBMCs) with the hydroalcoholic extract from aerial parts of *P. oleracea* (100 *μ*g/m) decreased the concentrations of TNF-*α* and IL-6 [59].

The results of the abovementioned (*in vitro*) studies indicated that *P. oleracea* extracts or fractions could inhibit production of cytokines as well as cytokine-induced ROS production. The plant extracts or fractions also inhibited TNF-*α*-induced degradation of cellular proteins and/or phosphorylation of some mitogen-activated protein kinases in the cells. *P. oleracea* extracts also increased the expression of membrane proteins in the immunoglobulin superfamily such as CD80, CD83, and CD86 that enhanced and sustained T-cell activation. Moreover, the plant fractions also increased expression of the conserved receptors (TLR-4) that recognize conserved pathogen-associated molecular patterns (PAMPs), thus representing the first line of defense against infections.

#### 4.1.2. *In Vivo* Studies

It was shown that various concentrations of *P. oleracea* polysaccharides decreased weight of the spleen and stimulated T and B lymphocytes in Wistar rats as dose dependently [60].

In D-galactose-induced aging mice model, *P. oleracea* polysaccharide (POP) showed preventive effects as decrease in the weight of the spleen and reduction in the number of spleen T cells at 30 days after D-galactose administration in mice [61].

In ovalbumin (OVA)-induced asthmatic rats, administration of drinking water contains hydroethanolic extract of *P. oleracea* decreased total protein (TP), phospholipase A2 (PLA2), and immunoglobulin E (IgE) levels in the bronchoalveolar lavage fluid (BALF) dose dependently [62]. In another similar study, the extract of *P. oleracea* and alpha-linolenic acid (ALA) significantly reduced NO_2_ and NO_3_ levels and total WBC count in serum. Furthermore, *P. oleracea* and ALA significantly increased lymphocyte percentages, while decreased the neutrophil and eosinophil percentages [63].

Oral administration of *P. oleracea* in LPS-induced acute lung injury (ALI) rats suppressed lung inflammation via the decline of IL-1*β*, IL-6, TNF-*α*, prostaglandin E2 (PGE2), and transforming growth factor beta (TGF-*β*), while increased IL-10 levels in the BALF. The *P. oleracea* extract also improved the level of WBC and myeloperoxidase (MPO) compared to the LPS group [64].

Lee et al. showed that treatment of diabetic mice with aqueous extract of *P. oleracea* (300 mg/kg/day, 10 weeks) increased the expression of intercellular adhesion molecule-1 (ICAM-1) and TGF-*β*1 in the renal cortex of untreated mice compared to treated ones [65]. Also, *P. oleracea* significantly suppressed the activation of NF-*κ*B p65 in renal tissues, which were increased in untreated mice [65]. In another study, the extract of *P. oleracea* (300 mg/kg/day) significantly suppressed overexpression of vascular cell adhesion molecule-1 (VCAM-1), ICAM-1, E-selectin, endothelin-1 (ET-1), and matrix metalloproteinase-2 (MMP-2) in aortic tissues of untreated mice [66].

The effects of anti-inflammatory of aerial parts of *P. oleracea* were shown on acute paw edema created by formalin in male mice [67].

In rats with chronic constriction injury (CCI), intraperitoneal use of *P. oleracea* considerably attenuated pain-related behaviors and contents of TNF-*α* and IL1*β* as dose dependently [68].

Pretreatment of rats with *P. oleracea* (400 mg/kg, p.o.) for 14 days in the LPS-induced neuroinflammation model improved the memory and reduced the level of TNF-*α* [69].

Inflammation was decreased via reducing proinflammatory genes including TNF-*α* and TGF-*β* in bile duct ligation-induced rat model of acute liver injury after treatment with 500 mg/kg methanolic extract of *P. oleracea* for 7 days [70]. Pretreatment of mice with 1, 2, and 4 g/kg aqueous extract of *P. oleracea* in a liver injury model induced by carbon tetrachloride (CCl4) decreased TNF-*α*, IL-1b, and IL-6 in serum [71].

In a IL-10-deficient mouse model, treatment of piroxicam-accelerated colitis IL-10-deficient (PAC IL-10^−/−^) mice with 400 mg/kg/day *P. oleracea* extract and proinflammatory parameters such as TNF-*α*, IL1*β*, and nuclear factor-kappa B (NF-*κ*B) were reduced which confirm anti-inflammatory effects of the extract [72]. Similarly, administration of a diet supplemented with 8% purslane plus 100 mg/l CdCl_2_ in water for 8 weeks inhibited inflammation via suppressing some cytokines (TNF-*α*, IL-6, IL-1*β*, and IFN-*γ*) in the colon of mice [73].

Oral gavage of the hydroethanolic extract of *P. oleracea* (100 and 300 mg/kg/day) in streptozotocin-induced type-I-diabetes rats for 8 weeks significantly improved MDA, TNF-*α*, and TGF-*β*1 as well as histopathological injury [74].

The effect of aqueous and ultrasound-assisted ethanol extracts of *P. oleracea* (3 mL 1 g/mL, twice a day for 3 weeks) on 2,4-dinitrochlorobenzene (DNCB)-induced atopic dermatitis mice was compared. Both extracts inhibited inflammatory factors including TNF-*α*, IFN-*γ*, and IL-4. Therefore, this plant can be regarded as an anti-inflammatory medicinal herb [75].

Surgical-induced peritoneal adhesion was improved after treatment of rats with hydroethanolic extract of *P. oleracea* (100 or 300 mg/kg/day, orally, for 7 days) through reduction of inflammatory factors (IL-6, TNF-*α*, and IL-1*β*) and enhanced anti-inflammatory cytokine (IL-10) [76].

The results of *in vivo* studies indicated that *P. oleracea* extracts showed anti-inflammatory properties due to a reduction in some enzymes and proteins that promote inflammation in mammals including total protein, PLA2, and IgE levels.

*P. oleracea* extracts also improved the levels of WBC and leukocyte-derived enzyme that catalyses the formation of a number of reactive oxidant species (ROS), as well as reduced the proinflammatory cytokines while increasing anti-inflammatory mediators. The plant extracts suppressed overexpression of the superfamily of proteins including VCAM-1, ICAM-1, ET-1, and MMP-2 that controlled a large variety of physiological and pathological processes, including tissue remodelling, DNA replication, cell-cycle progression, and cancer.

### 4.2. Anti-Inflammatory Effects of the Plant Constituents

#### 4.2.1. Alpha Linolenic Acid

*(1) In Vitro Studies*. ALA is an essential omega-3 fatty acid [77]. ALA is also known as one of the important components of *P. oleracea* [78]. It is demonstrated that ALA regulated the immune system by acting on T lymphocytes [79].

Treatment of LPS-stimulated human corneal epithelial (HCE) cells with ALA (125 *μ*M) significantly ameliorated the stimulation-induced increase in mRNA and protein levels of TNF-*α*, IL-1*β*, IL-6, and IL-8 [80]. The anti-inflammatory activity of ALA on proinflammatory cytokines was similar to that of dexamethasone (10−5 M). The inhibitory activity of ALA on the proinflammatory cytokines was connected with a reduction in inhibitory factor *κ*B*α* (I-*κ*B*α*) [80].

Preincubated human monocytic THP-1 cells\ with ALA (100 *μ*M) for 2 h ameliorated the effect of 24 h LPS (1 *μ*g/ml) incubation. ALA pretreatment significantly decreased secretion of IL-6, IL-1*β*, and TNF*α* and also reduced the mRNA levels of IL-6, IL-1*β*, and TNF**α** compared to the LPS treated cells [81]. In addition, ALA treatments considerably suppressed the nuclear factor (NF)-*κ*B DNA-binding activity which was increased by LPS treatment [81].

The preventive effect of ALA (5 and 10 mg/kg) on the stimulatory action of LPS-induced NO production was reported in macrophages' cell of mice (RAW 264.7). ALA also inhibited LPS stimulatory action on TNF-*α*, iNOS, and cyclooxygenase-2 (COX-2) gene expressions [82]. ALA treatment reduced the NF-*κ*B-dependent transcriptional activity and translocation of the NF-*κ*B subunit. Furthermore, ALA suppressed phosphorylation of mitogen-activated protein kinases (MAPKs) [82].

In hypercholesterolemic subjects, the production of proinflammatory cytokine by cultured peripheral blood mononuclear cells (PBMCs) had been reduced after high ALA (6.5% of energy) supplementation [83]. ALA also reduced IL-6, IL-1*β*, and TNF-*α* by PBMCs. In addition, TNF-*α* production by PBMC was inversely correlated with ALA use [83]. These results indicated anti-inflammatory effects of ALA by reduction of the production of inflammatory cytokines.

*(2) In Vivo Studies*. In a mice model of neuroinflammation induced by cadmium, oral administration of ALA (60 mg/kg) for 6 weeks suppressed NF-*κ*B and IL-1*β* in the brain tissue, which suggests neuroprotective effects of ALA [84]. Leung et al. reported a decrease in the levels of inflammatory factors such as IL-1*β*, IL-6, and TNF*α* following treatment of mice with ALA-enriched diets for 28 days [85].

In a mouse model of OVA-induced allergic rhinitis, treatment of animals with ALA (500 and 2000 mg/kg, daily, for 13 days) reduced expression of IL-6 and IL-1*β* in nasal mucosa that show anti-inflammatory effects of this essential fatty acid [86].

Dietary supplementation with ALA (8 g) in dyslipidemic patients (76 male, mean age = 51 ± 8 years) for 3 months significantly decreased IL-6, amyloid A (SAA), and C-reactive protein (CRP) levels in serum [87]. The authors concluded that decrease in inflammatory cytokines was independent of lipid changes.

#### 4.2.2. Quercetin

*(1) In Vitro Studies*. Quercetin is a polyphenolic flavonoid with anticancer, antioxidant, and antiviral features which are found in several plants such as *P. oleracea* [88]. Quercetin suppressed TNF-*α* generation as dose dependently and impaired chemokines and cytokines levels in DCs stimulated by LPS. In addition, quercetin significantly reduced production of cytokines and chemokines in LPS-stimulated DCs [89]. Also, quercetin suppressed enhanced expressions of CD40, CD80, and CD86 in LPS-stimulated DCs. The cytokines secreted by activated DCs were downregulated by quercetin, indicating the immunoregulatory function of this agent on DCs [89].

In primary cells, quercetin (40 *µ*M) suppressed generation of IFN-*γ* and IL-2 with T-cell receptor (TCR) stimulation. Quercetin significantly inhibited the increase of IL-2Ra expression, when TCR and exogenous recombinant human IL-2 are stimulated [90].

Treatment with quercetin (0.5–50 *μ*M)) increased IFN-*γ* gene expression and production but downregulated IL-4 in normal PBMC (1 × 106 cells/ml) [91]. Quercetin (10, 25, and 50 *µ*M) significantly increased IFN-*γ* expression in PBMC cultures supernatant; however, quercetin (0.5–50 *μ*M) significantly suppressed IL-4 expression and markedly reduced its secretion in PBMC cultures' supernatant [91].

Quercetin alone and combined with interferon beta (IFN-*β*) (quercetin 50 *µ*M + IFN-*β* 2IU/ml) reduced PBMC proliferation and IL-1*β* and TNF-*α* production. The mixture of quercetin and IFN-*β* indicated augmentative effects in MMP-9 and TNF-*α* modulation [92]. Moreover, quercetin declines the ratio of MMP-9/tissue inhibitor of metalloproteinases-1 (TIMP-1) by dose dependently reducing the MMP-9 generation [92].

Quercetin (10–30 *μ*mol/L) protected human umbilical vein cells (HUVEC) culture versus lipid peroxidation induced by H_2_O_2_. Quercetin also decreased the NF*κ*B transcriptional activity and cell-surface E-selectin and VCAM-1 expression induced by cytokines [93].

Administration of quercetin (1, 10, and 50 *µ*M) suppressed expressions of iNOS, TNF-*α*, IL-1*β*, and IkB-*α* phosphorylation induced by LPS and macrophage colony-stimulating factor (M-CSF). Also, treatment with 1 mg/kg/day quercetin inhibited dextran sulphate sodium (DSS)-induced expression of IL-1*β* and TNF-*α* and iNOS in rats [94].

*(2) In Vivo Studies.* Administration of quercetin (0.1%, w/w) with standard chow for 14 days in mice-attenuated atherosclerosis and vascular inflammation including IL-1R, chemokine (C-C motif) ligand 8 (CCL8), and I*κ*B kinase (IKK) also reduced inflammatory risk factors' concentrations in plasma, serum amyloid A, and fibrinogen [93].

Quercetin (8 and 16 mg/kg/day) administration significantly reduced the BALF level of eosinophils (68.79% and 73.35%, respectively) in mice challenged with OVA [95]. Quercetin also decreased IL-4 and IL-5 secretion, as well as MMP-9 and erythropoietin (Epo) mRNA expression but increased the IFN-*γ* concentration in the BALF compared to nontreated animals [95].

Treatment with quercetin (500, 1000, and 1500 mg/day, p.o) or coadministration with azathioprine (100 mg/day) for eight weeks in rheumatoid arthritis patients compared to the placebo was investigated [96]. Coadministration of high dose of quercetin significantly declined the IL-6 and complement (C3 and C4) but elevated the level of IL-10 compared to the placebo-treated group. Treatment with different doses of quercetin significantly reduced ICAM-1 compared to the azathioprine alone-treated group [96].

Oral gavage of 50 mg/kg quercetin in mice with inflammatory bowel diseases (IBD) led to reduce of Th17 cells but increase in the number of Treg cells, resulting in reduction of gut inflammation [97].

**Figure 2:**
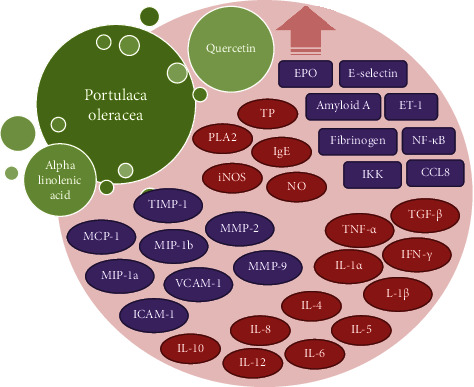
A summary of the possible mechanisms of anti-inflammatory effects of *P. oleracea* and its constituents. IL: interleukin; NO: nitric oxide; iNOS: inducible nitric oxide synthase; MCP: monocyte chemoattractant protein; TIMP-1: tissue inhibitor of metalloproteinases-1; CCL8: chemokine (C-C motif) ligand 8; IKK: I*κ*B kinase; TNF-*α*: tumor necrosis factor-alpha; TP: total protein; PLA2: phospholipase A2; TGF-*β*: transforming growth factor beta; MMP: matrix metalloproteinase; ET-1: endothelin-1; EPO: erythropoietin; ICAM-1: intercellular adhesion molecule-1; VCAM-1: vascular cell adhesion molecule-1; IgE: immunoglobulin E.

These results indicated that *P. oleracea* and its components showed anti-inflammatory properties and promote subsets of T-cell toward T-helper1 (IFN-*γ*) and Treg secretion (IL-10), which may indicate a therapeutic use of the plant and its constituents for the treatment of inflammatory and allergic diseases. In addition, quercetin and ALA (the constituent of the plant) inhibit inflammatory cytokine levels and downregulate secretion of cytokines and chemokines. Thus, *P. oleracea* and its active components could be potentially used for prevention and therapy of inflammatory and allergic disorders such as COPD and asthma.  Table 2 shows anti-inflammatory properties of *P. oleracea* and its components. In addition, possible mechanisms of the anti-inflammatory activity of the plant and its major components are summarized in Figure 2.

## 5. Antioxidant Effects

The antioxidant activity of natural products was reported by several studies [99–104]; therefore, these compounds could be considered as good candidates for cytoprotective agents. Glutathione reductase (GR), glutathione-S-transferase (GST), glutathione peroxidase (GPx), glutathione (GSH), catalase (CAT), and superoxide dismutase (SOD) play a pivotal role for oxidative stress defense [105, 106]. Various clinical studies showed that glutathione acts as a detoxifying and antioxidant agent [107–113]. Glutathione is a substrate for GPx and thereby reduces lipid peroxides. It also acts as a glutathione-S transferase which conjugates electrophilic compounds. Dietary glutathione is absorbed via the gastrointestinal system and improved human's antioxidant status [114].

### 5.1. Antioxidant Effects of *P. oleracea*

#### 5.1.1. *In Vitro* Studies

Antioxidant properties and mineral composition of *P. oleracea* were evaluated at several growth steps. The antioxidant capacity was evaluated by 1,1-diphenyl-2-picryl-hydrazyl (DPPH) and ferric-reducing antioxidant power (FRAP) assays [115]. Mature plants of *P. oleracea* had more total phenol content (TPC) and antioxidant effects than plants at the immature steps. Particularly, 60-day-old plants had large amounts of TPC and antioxidant capacity as evaluated by the DPPH test and FRAP assay [115].

*P. oleracea* aqueous extracts obtained from leaves, stems, and flowers were examined for the total phenolic content, antioxidant activity, and ferric-reducing antioxidant power [116]. Significantly, higher values were observed for stems than in flowers and/or leaves. In the DPPH assay, the 50% inhibition rate is obtained in lesser concentrations for the three plant parts [116].

*In vitro and in vivo* studies were done to assess antioxidant capacity of *P. oleracea* leaves' ethanolic extract. The extract (25, 50, and 100 mg/kg) considerably decreased lipid peroxidation and remade the nonenzymatic and enzymatic antioxidants levels in the liver tissue. The *P. oleracea* ethanolic extract at a dose of 100 mg/kg was more effective than doses of 25 and 50 mg/kg [117].

The *in vitro* antioxidant effect of *P. oleracea* methanolic extract was investigated by different methods including NO free radical scavenging activity, DPPH free radical scavenging activity, superoxide scavenging activity, reducing power by FeCl, and the alkaline DMSO method. The *in vitro* antioxidant activity of *P. oleracea* methanolic extract was showed to be more than standard antioxidants [118].

The total phenol contents of six cultivars of *P. oleracea* methanolic extracts had been analyzed by the Folin–Ciocalteu method. There was a well relation between the total phenol content value and its ascorbic acid equivalent antioxidant capacity and FRAP values in all of the cultivars [119]. The ascorbic acid content for the cultivars was from 38.5 ± 0.6 to 73.0 ± 17.5 mg/100 g. The BCB assay demonstrated that all cultivars were able to inhibit lipid peroxidation, and the inhibitory effect did not relate to the total phenol content value [119].

The *in vitro* antioxidant effect of leaves of *the P. oleracea* methanolic extract from Turkey was evaluated by DPPH and *β*-carotene-linoleic acid assays. The extract exhibited high levels of free radical scavenging activity [120].

Radical scavenging activities were measured for evaluating the solvent extracts of *P. oleracea*. The electron-donating abilities (EDAs) of ethyl acetate and methanolic extracts showed high antioxidant activity. The SOD-like abilities of ethyl acetate and petroleum ether extracts also showed some activities. However, there was not a significant antioxidant activity for the thiobarbituric acid reactive substances [121].

Total antioxidant capacity of fresh and dried hydroalcoholic *P. oleracea* extracts was measured using radical cation (ABTS) and DPPH tests and the FRAP assay. Fresh hydroalcoholic *P. oleracea* extract showed the highest radical scavenging power in ABTS and DPPH tests [122].

It was shown that different drying methods (microwave drying, hot-air drying, and freeze-drying) may affect the antioxidant capacity of *P. oleracea* leaves. The best antioxidant activity was seen for fresh purslane leaves as 147.78 *μ*mol trolox and 53.23% per 100 g dry weight for ABTS and ABTS, respectively [123]. Between dried samples, those dehydrated by freeze-drying and different hot-air drying temperatures method showed maximum antioxidant capacity and no significant differences was observed between both ABTS and DPPH methods [123].

Total phenolic content and antioxidant capacity of the fractions of *P. oleracea* crude extract which were obtained using reversed-phase separation method were determined. According to optical absorption, five fractions were isolated [124]. In comparison to crude extract, the quantified total phenolics amount in fraction 3 was higher. In fraction 3, free phenolic acids including caffeic, chlorogenic, ferulic, p-coumaric, and rosmarinic acids were detected, and also free flavonoids (quercetin and kaempferol) were determined too. Trolox equivalent antioxidant capacity (TEAC) of the crude extract was four times lower than fraction 3 TEAC. The thiobarbituric acid reactive substance (TBARS) assay method showed the highest lipid peroxidation inhibition activity for fraction 3 [124].

#### 5.1.2. *In Vivo* Studies

The antioxidant activity aqueous juice from *P. oleracea* was determined by measuring reduced GSH, CAT, SOD, GR, GST, and GPx, as well as NO and lipid peroxidation inhibition in the kidney, liver, and testis of rats [114]. Aqueous juice of *P. oleracea* (1.5 ml/kg, orally) administration in rats resulted in marked improvement in parameters related to kidney and liver function [114]. Water extract of *P. oleracea* decreased the serum and lipid peroxidation levels in the liver and increased the antioxidant enzymes activities in the liver and serum in high-fat mice [125]. Pretreatment with the *P. oleracea* aqueous extract (300 mg/kg) prevented the lactate dehydrogenase (LDH), alanine aminotransferase (ALT), and alkaline phosphatase (ALP) increased in the nephrectomized rat model subsequently ischemia-reperfusion (IR) injury in the kidney [126].

Ethanolic and aqueous extracts of *P. oleracea* showed cytoprotective effects in free radical initiator and AAPH (‘2, 2′ azobis (2-amidinopropane) hydrochloride)-induced damages [127]. The ethanolic and aqueous extracts of *P. oleracea* decreased the rate of AAPH-induced hemolysis. The plant increased the lag time of AAPH-induced hemolysis and decreased RBC damages [127].

The protective effect of *P. oleracea* ethanolic extract versus hepatic toxicity induced by carbon tetrachloride (CCl4) was investigated. Intragastric administration of the *P. oleracea* extract normalized hepatic marker enzymes in rats treated to CCl4. Both liver histopathological alterations and marker of liver function improved after *P. oleracea* treatment [128].

The antioxidant activity of *P. oleracea* (POEE, 4 mg/kg, orally, for 21 days) versus the MeHg-induced neurotoxicity (5 mg/kg for 21 days) was investigated in the cerebellum and cortex of rats [129]. The activities of CAT, SOD, GPx, and the level of GSH were declined but GR and malondialdehyde (MDA) levels were enhanced in cerebellum and cortex by MeHg. All of biochemical changes were reversed after POEE treatment [129].

Treatment with fresh and dried *P. oleracea* in C57BL/6J diabetic mice significantly decreased the MDA level and enhanced SOD activity in the liver relative to untreated diabetic mice [130].

The effect of *P. oleracea* on the oxidant-antioxidantcondition in hepatic toxicity induced by paracetamol was evaluated in rats. Paracetamol induces toxic effect including depletion in total antioxidant capacity, an increase in the hepatic TBARS content, and reduction in GSH content, catalase, and superoxide dismutase activities. Coadministration of *P. oleracea* (300 mg/kg, orally) and paracetamol significantly prevented the hepatotoxicity of paracetamol [131].

In a study, ameliorative effects of 1, 2, and 4 mg/mL *P. oleracea* extract on oxidative biomarkers including MDA, SOD, CAT, and thiol were observed in the BALF of asthmatic rats [132].

Saleh et al. showed that administration of ethanolic extract of *P. oleracea* (100 mg/kg) and Chicory water extract (100 mg/kg) in rats with testicular and autophagy dysfunction synergetically improved testicular toxicity via reducing MDA and enhancing GSH, GST, and GPx activities [133]. Similarly, in the rat model of testicular toxicity induced by acrylamide, oral administration of 200 and 400 mg/kg *P. oleracea* extract improved testicular toxicity through reduction of oxidative status [134].

Antioxidant effect of the hydroalcoholic extract of *P. oleracea* (25, 50, and 100 mg/L) on functional parameters of human sperm samples was evaluated. Reduction of intracellular ROS and increase of motility of sperm were observed after treatment [135].

Treatment with hydroalcoholic extract of *P. oleracea* (400 mg/kg) in a thyrotoxic rat model improved hyperthyroid state by reducing MDA level and increase of thiol, SOD, and CAT activities [136].

Based on the abovementioned experimental studies, *P. oleracea*-based extracts showed antioxidant effects by increasing antioxidant markers such as CAT, GSH, and SOD and decreasing oxidant parameters including MDA and NO levels.

### 5.2. Antioxidant Effects of the Plant Constituents

#### 5.2.1. Alpha Linolenic Acid

*(1) In Vitro Studies*. Antioxidant activity of plant sterol ester of *α*-linolenic acid (PS-ALA) was examined using the *in vitro* model. In this model, HepG2 cells induced by oleic acid treated to 0.1 mM ALA-PS for 24 h. The results showed reduction of ROS production in oleic acid-loaded HepG2 cells after treatment with PS-ALA [137].

*(2) In Vivo Studies.* Oxidative stress agents and antioxidative enzymes were evaluated in rats fed with a single lipid source such as sunflower oil, canola oil, rosa mosqueta oil, sacha inchi oil, and chia oil containing different amounts of ALA [138]. The results showed that higher supply of ALA improved the antioxidative status (GSH content and GSH/GSSG ratio as well as the antioxidant enzyme activity such as SOD, CAT, GPx, and GR) [138].

The antioxidant effect of ALA on oxidative stress induced by amyloid-beta peptide was evaluated in a rat model. Treatment with 150 *μ*g/kg ALA for 2 weeks reduced MDA and NO levels but increased CAT activity and glutathione content in hippocampus [139].

#### 5.2.2. Quercetin

*(1) In Vitro Studies*. The cytoprotective effect of quercetin was examined on iron-loaded hepatocyte cultures. In the presence of quercetin, the amount of MDA was decreased as dose dependently [140]. The damaging effect of Fe-NTA on hepatocytes led to increase of LDH in the culture medium and a decrease in intracellular LDH levels. Permeation of enzyme was inhibited using quercetin as dose dependently [140].

The influence of the chemical structure on antioxidant capacity of quercetin was evaluated by examining of inhibition of copper-catalysed oxidation of human low density lipoprotein (LDL) *in vitro*. Quercetin (2.5, 5, and 7.5 *µ*M) inhibited human LDL oxidation in a dose-dependent manner [141].

Erythrocyte membranes versus lipid peroxidation were protected by quercetin through inhibition of hemolysis and GSH depletion [142].

In a study, mouse thymocyte was used to investigate whether quercetin acted as an antioxidant or as a cytotoxic factor. Quercetin protected thymocytes against apoptosis induced by glucose oxidase in mice as dose dependently [143]. In addition, electrophoretic mobility shift assays (EMSAs) showed that 50 *µ*M quercetin inhibited the glucose oxidase-mediated DNA-binding activity of redox state sensitive transcription factors, such as NF-kappaB, AP-1, and p53. These results indicate antioxidative effects of quercetin on thymocytes [143]. Quercetin (10, 20, 50, 100, or 1000 *µ*M) decreased the formation of hydroperoxides from methyl linoleate as concentration dependently [144].

The quercetin antioxidant effect was assessed by two methods of measuring the ability to scavenge the ABTS radical cation at different pH and also FRAP assay. In these tests, the quercetin acts as radical scavenger and decrease compound antioxidant agents as an *α*-tocopherol [145]. It was also shown that antioxidant activity on antisuperoxide formation, DPPH scavenging, antilipid peroxidation, and superoxide anion scavenging was less potent in pure quercetin than the quercetin-loaded nanoparticles [146].

Moreover, the antioxidant capacity of the quercetin-nanoparticles *in vitro* using reducing power test and free radical scavenging activity test showed that quercetin-loaded nanoparticles in the DPPH test reduced the stable radical DPPH, and its reducing power was correlated well with increasing concentration [147].

*(2) In Vivo Studies.* In rats adapted for 3 weeks to a semipurified diet riched with 0.2% quercetin, it was shown that the total antioxidant status of plasma in the group fed with the quercetin was significantly more than that in the control group [148]. Treatment of streptozotocin (STZ)-induced diabetic rats with 15 mg/kg, quercetin reduced MDA and NO levels and also increased the GSH-Px, SOD, and CAT activities [149]. The protective effect of quercetin (75 mg/kg) was also shown in a rat model of cyclophosphamide-induced hepatotoxicity. Treatment of animals with quercetin for 10 days decreased oxidative stress parameters including MDA and protein carbonyl (PCO) [150]. In liver injury induced by bile duct ligation, treatment of rats with 50 mg/kg/day quercetin (for 10 days) reduced oxidative stress through suppressing the oxidation of proteins and activity of the glutathione peroxidase [151].

It was shown that the plasma antioxidant status was markedly upper in animals treated with quercetin (50 mg/kg, intragastrically), [152]. Subacute treatment with quercetin treatment (10 mg/kg) in STZ-induced diabetic rats ameliorated the effects of diabetes on hepatic GPx activity and brain oxidized GSH concentration. There was 40% decline in GSH concentration of liver, 20% increase in the liver MDA level, and increase in GPx activities of renal (23%) and cardiac (40%), as well as a 65% increase in CAT activity of cardiac tissue. These results showed the preventive effects of quercetin in oxidative stress-induced diabetes [153].

Antioxidant and antiulcerogenic effects of quercetin in gastric lesions induced by ethanol in rats showed significant decreased gastric injury and MDA level and significantly enhanced GSH-Px, SOD, and CAT activities [154].

In a model of tramadol intoxication, rats received 100 mg/kg of quercetin for 14 days. Findings showed improvement of oxidant/antioxidant agents (MDA, SOD, and NOx) in various tissues including the heart, liver, adrenal, and kidney [155]. In another study, protective effect of quercetin was shown in a rat model of hepatotoxicity and renal toxicity induced by radiation. Oral administration of 50 mg/kg quercetin before or after radiation led to decrease of MDA level in liver and kidney tissues [156].

Pretreatment of mice exposed to cigarette smoke with quercetin (10 mg/kg/day) improved pulmonary function and emphysema via reduction of inflammatory cytokines, increase of SOD and CAT activities, and decline of myeloperoxidase activity which suggest that quercetin may be used as an antioxidant agent [157]. In addition, the effect of quercetin on expression of antioxidant genes including CAT, superoxide dismutase 1 (SOD1), glutathione peroxidase 1 (GPX1), and antioxidant capacity (TAC) was evaluated in the dental pulp of diabetic rats induced by streptozotocin. Oral gavage of quercetin (25 mg/kg) for 40 days improved CAT, SOD1, GPX1, and TAC levels [158].

#### 5.2.3. Other Constituents

The antioxidant activity of *P. oleracea* seed oil (PSO) was evaluated using different methods *in vitro*. The higher concentrations of PSO (4 mg/ml) elevated hydroxyl free radical scavenging activity. Increase in the oil concentration improved the DPPH scavenging ability [159]. There was a linear relationship between scavenging ability and PSO concentrations at the range of 3–20 mg/ml. Moreover, antioxidant activity of PSO detected at concentrations ≥3 mg/ml. Moreover, the TBHQ free radical scavenging capacity of PSO was more potent than that of DPPH [159].

The evaluation of the antioxidant capacity of the various extracts of *P. oleracea* leaves using two methods of extraction including hot-maceration and rapid solid-liquid dynamic extraction showed a inhibition percentage of 52% and 54%, respectively, while the mixed extraction method showed a high inhibition rate (70%) [160].

The antioxidant activities of polysaccharide fractions purified from *P. oleracea* were examined by cell-free and cell-mediated radical generating systems. The fractions possessed strong antioxidant activities in both systems [161].

The antioxidant potential of an oral treatment (3 weeks) of diabetic rats with *P. oleracea* polysaccharide fraction (PPFt) (25 and 50 mg/kg) dose dependently reduced the serum TBARS and glucose and increased in total GPx activity and GSH levels. PPFt administration also improved SOD and CAT values near to optimal levels [162].

Antioxidant activities of phenolic alkaloids extracted from *P. oleracea*, such as oleracein A (OA), oleracein B (OB), and oleracein E (OE), were indicated. The inhibitory effects of these compounds on lipid peroxidation in brain tissue in rats were evaluated [41]. These phenolic alkaloids exhibited lower DPPH radical scavenging activities than caffeic acid but their DPPH radical scavenging were higher than ascorbic acid and *α*-tocopherol (OB > OA > OE). OE preventing effects for lipid peroxidation was most potent with an EC50 of 73.13 *µ*M, near to caffeic acid (72.09 *µ*M). It was indicated that phenolic alkaloids served as new antioxidant factors in this plant [41].

In a study, the antioxidant effect of *P. oleracea* was examined in a vitamin A deficiency induced-oxidative stress rat model. Feeding rats with pure beta-carotene diet or a diet supplemented with *P. oleracea* leaves resulted in lower TBARS concentrations in liver and heart tissues compared to vitamin A-deficient diet rats [163]. The liver GSH and heart glutathione disulfide (GSSG) concentrations of the *P. oleracea* treated group were lower than vitamin A-deficient group [163].

**Figure 3:**
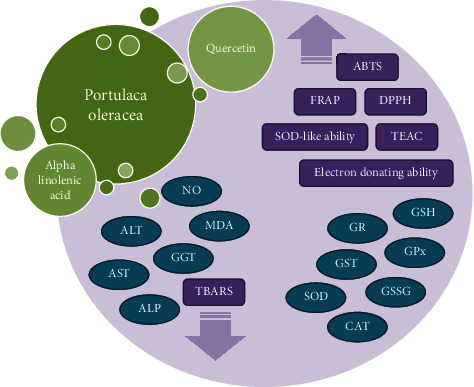
Possible mechanisms of antioxidant effects of *P. oleracea* and its constituents. ABTS: antioxidant capacity determined by radical cation; FRAP: ferric-reducing antioxidant power; TBARS: thiobarbituric acid reactive substances; DPPH: 1,1-diphenyl-2-picryl-hydrazyl; TEAC: Trolox equivalent antioxidant capacity; CAT: catalase, SOD: superoxide dismutase, MDA: malondialdehyde; GSH: glutathione; GPx: glutathione peroxidase; GR: glutathione reductase; ALT: alanine aminotransferase; AST: aspartate aminotransferase; ALP: alkaline phosphatase; GGT: gamma-glutamyl transferase; GSSG: glutathione disulfide.

The antioxidant enzymes, including GPx, GR, GST, SOD, and CAT, have an important role in maintaining glutathione homeostasis in tissues [39]. Enhanced levels of GPx, GR, GST, SOD, and CAT were related to increased glutathione level and depressed MDA, NO, and lipid peroxidation in the liver, kidney, heart, and testis of rats, thus indicating the antioxidant activity of *P. oleracea*. The reduction of the liver enzymes activity, ALT, AST, *γ*-GT, and ALP in the *P. oleracea* treatment group indicates its protective role versus liver injury [39]. The antioxidant effects of *P. oleracea* and its constituents are summarized in Table 3.  Figure 3 also shows possible mechanisms of antioxidant effects of *P. oleracea* and its components.

Abovementioned *in vitro* and *in vivo* studies showed that *P. oleracea* constituents, especially quercetin, have the potential to be used as an antioxidant agent. This claim is proved by increasing FRAP, ABTS, and DPPH scavenging capacity and decreasing MDA concentration, superoxide and hydroperoxide anions, and lipid peroxidation. However, further clinical studies regarding the effect of *P. oleracea* and its constituents are needed.

## 6. Immunomodulatory Effects

Immunomodulation is the modulation of the function of the immune system or an immune response with use of a drug or herbal compound. Immunomodulatory drugs change immune response by the stimulation (immunostimulators) or inhibition (immunosuppressives) of antibody formation or the white blood cell activity [164, 165]. Many plants and their bioactive components were studied for their possible use as immunomodulation drug [166, 167].

### 6.1. Immunomodulatory Effects of *P. oleracea*

#### 6.1.1. *In Vitro* Studies

Aqueous alcoholic extract of *P. oleracea* on T helper cells Th1/Th2 balance in stimulated and nonstimulated human lymphocytes showed that the extract (160, 40, and 10 *µ*g/ml) reduced cell proliferation and serum level of cytokines but enhanced IFN-*γ*/IL-4 ratio and Treg/Th2 (IL-10/IL-4) balances in stimulated cells [54]. The results were compared to control and dexamethasone as positive control and showed immunomodulatory effects of plant which were lower than dexamethasone [54].

Treating BALB/c-isolated splenocytes stimulated by concanavalin A (Con A) indicated that ethyl acetate and chloroform extracts of *P. werdermannii* and *P. hirsutissima* inhibited the proliferation of cells suggesting immunomodulatory activity of this plant [168]. In this study, extracts from aerial parts of *Portulaca* were tested and more studies to identify the active compounds present in these extracts for the treatment of immune-mediated the disorders are needed [168].

#### 6.1.2. *In Vivo* Studies

Immunomodulatory evaluation ethyl acetate extract of *P. oleracea* in cyclophosphamide-treated mice showed increased phagocytosis and elevated proliferation of splenic lymphocytes due treatment with of *P. oleracea* suggesting immunoactivity property of the plant [169]. However, there are no data about the mechanisms by which the active compounds present in the plant could induce immunomodulatory effects.

Immunomodulatory effect of *P. oleracea* extract on IL-4 and interferon gamma (IFN-*γ*) and IFN-*γ*/IL4 ratio in BALF of asthmatic rats showed an immunomodulatory effect through increase of IFN-*γ*/IL4 ratio [170]. The effect of administration of *P. oleracea* extract on immune markers in asthmatic rats during sensitization period showed reduction of BALF levels of IgE indicating modulation of immune function [62].

Barakat and Mahmoud reported that purslane/pumpkin seed mixture has immunomodulator effects on hypercholesterolemic rats through improvement of immunoglobulin G (IgG) and immunoglobulin M (IgM) levels, which is probably due to present of ALA in the mixture [171]. Oral administration of extract (200 mg/kg) for 7 days in a mice model of acetic acid-induced ulcerative colitis significantly reduced IL-1, IL-6, IL-17, TNF-*α*, IFN-*γ*, and nuclear factor-kappa B as well as myeloperoxidase activity. The findings suggest immunomodulatory property of the ethanolic extract of *P. oleracea* [172].

In a cyclophosphamide-induced immunosuppressive rat model both *in vivo* and *in vitro* (isolated spleen cells), immunostimulatory effects of *P. oleracea* L. and Perilla frutescens seed complex extracts (PPCE) were evaluated [173]. The findings showed that treatment with PPCE led to increase of immune cells and splenic recovery. The PPCE also enhanced proliferation of splenocyte and inflammatory cytokines including IL-2, IL-12, TNF-*α*, and IFN-*γ* as well as NK cell activity. According to these results, *P. oleracea* could be used as an immunomodulatory agent [173].

Ethyl acetate and ethanolic extracts of *P. oleracea* on inflammatory responses induced by LPS in RAW264.7 macrophages (*in vitro* model) and dextran sulphate sodium (DSS)-induced colitis in mice (*in vivo* model) indicated the inhibition of the serum proinflammatory cytokines (TNF-*α*, IL-6, and 1L-1*β*) in both models. According to these findings, *P. oleracea* can appropriately regulate immune responses [174].

Administration of 20 mg/ml *P. oleracea* in mice with atopic dermatitis led to decrease of serum level of Ig E and histamine HIS, reduction of inflammatory mediators such as TNF-*α*, IFN-*γ* and IL-4 as well as mast cell infiltration [175].

All *in vivo* studies were well designed and showed that *P. oleracea*-based extracts has the potential to be used as a potent immunomodulatory agent. However, more studies should be carried out in future to identify active compounds responsible for immunomodulatory effects of herb and its possible mechanisms.

### 6.2. Immunomodulatory Effects of the Plant Constituents

#### 6.2.1. Alpha Linolenic Acid

*(1) In Vivo Studies*. The effect of an fatty acids enriched diet on Th1/Th2 polarization in lymphocytes of mice showed significant increased ratio of IFN-*γ*/IL-4 in mice fed the omega-3 compared to omega-6. Thus, an omega-3 rich diet containing ALA induce a shift in Th1/Th2 balance and modulates immune function [176]. Increased serum IgG and IFN- *γ* as well as reduced IL-4 and PGE2 were observed in cows receiving 0, 100, 200, 300, and 400 g/d ALA by duodenal cannulas [11]. Thus, the results suggest immunomodulatory effects of ALA by modifying production of Th1/Th2 cytokines and the effect of plant on T-cell-mediated immunity. In this study, the infusion of large quantities of ALA and secretion into milk fat may affect immunity which needs more investigations [11].

Jeffery et al. [177] showed that feeding rats with a high-fat enriched diet (178 g lipid/kg) containing the ratios of palmitic, oleic, linoleic, and ALA for 6 weeks caused modifying the spleen lymphocyte functions [177]. Similarly, feeding weanling rats with high-fat diets contained 4.4 g ALA, *γ*-linolenic, ara-chidonic (ARA), eicosapentaenoic (EPA), or docosahexaenoic acid/100 g total fatty acids affects lymphocyte functions and cell-mediated immunity [178]. The finding indicated that replacing ALA with EPA in dietary leads to reduction of lymphocyte proliferation and natural killer (NK) cell activity as well as cell-mediated immune response [178]. The effect of ALA (0.2 and 0.4 mg/ml) on immune markers in sensitized rats with OVA also showed increase of IFN-*γ* and IFN-*γ*/IL4 ratio as well as decreased IL-4 indicating an immunoregulatory effect for the ALA [179].

The effect of alpha-linolenic acid on immune responses was shown in the abovementioned *in vitro* studies. However, further studies including clinical trials are needed to recommend it as immunoregulator.

#### 6.2.2. Quercetin

*(1) In Vitro Studies*. The immunomodulatory activity quercetin on human mesangial cells (HGMCs) showed reduction of nuclear factor-*κ*B p65 and IKK*β* expression, increased I*κ*B*α* expression, and inhibition of the expression of PTX3 antibody through inhibition of NF-*κ*B signalling pathway [180]. Further studies are needed to determine whether quercetin has inhibitory effects on PTX3 *in vivo*. Immunomodulatory activity of quercetin-3-O-*α*-L-rhamnopyranoside versus inflammatory reactions of influenza infection by propagation of influenza virus in Madin Darby–Canine kidney (MDCK) cells and incubation of quercetin-3-O-*α*-L-rhamnopyranoside (150 *μ*g/ml) for 48 h indicated reduction of TNF-*α* expression and increased IL-27 expression as pro and anti-inflammatory cytokines, respectively [181]. In model of asthma induced by Blomia tropicalis, treatment of spleen cells with 3.5, 7.5, and 15 *μ*g/ml quercetin for 48 h led to reduction of IL-4, IL-5, and IL-13 cytokines in spleen cell culture supernatants. These results suggest a potential therapeutic role of quercetin as an immunomodulatory agent [182].

In an *in vitro* model, immunomodulatory effect of incubation of PBMCs incubated with shikimic acid and quercetin at concentrations (10 and 100 nM) for 24 h showed that combination of shikimic acid and quercetin led to marked increase of IL-8 and IL-6 compared to baseline levels whereas incubation of cells with tamiflu did not change cytokine levels [183]. The results suggest the modulatory effect of shikimic acid and quercetin combination on innate immunity [183]. In mouse dendritic cells (DCs) treated with quercetin in the presence of LPS for 24 h, cytokines and chemokines (MIP-1a, MIP-1b, MCP-1, and RANTES) were reduced [89]. In addition, quercetin decreased the expression of CD40, CD80, and CD86 in DCs. These results show immunosuppressive property of quercetin on DCs activation and function [89].

The effect of different concentrations of quercetin (1, 10, and 50 *µ*M) on LPS-stimulated bone marrow-derived macrophages (BMDMs) indicated that treatment of cells with quercetin inhibited expression of high levels of TNF-*α* and IL-1*β* proteins, IkB-*α* phosphorylation, and iNOS expression induced by LPS in BMDM [94]. The peripheral blood mononuclear cells (PBMCs) of normal subjects incubated with 0.5–50 *µ*M quercetin for 24–72 h significantly enhanced IFN-*γ* gene expression and its secretion in cell supernatant while expression and generation of IL-4 markedly decreased by normal PBMC [91]. Therefore, induction of Th1-derived cytokines and inhibition of Th2 derived cytokines could be the possible mechanisms of immunomodulatory effects of quercetin [91]. Sternberg et al. showed that PBMC proliferation, the generation of cytokines, and matrix metallopeptidase 9 (MMP-9)/tissue inhibitor of metalloproteinases-1 (TIMP-1) ratio in the presence or absence of quercetin were evaluated [92]. Exposure of cells with 5–200 *µ*M quercetin for 48 h dose dependently reduced PBMC proliferation and cytokine levels in cell culture supernatants. Dose-dependent effect of quercetin on MMP-9/TIMP-1 ratios was also observed which suggest immunomodulatory effects of quercetin [92].

In an *in vitro* study, CD4+ T cells were derived from T-bet transgenic/deficient mice and incubated with anti-CD3 and anti-CD28 in the presence/absence of quercetin (40 *µ*M) for 24 h. Cytokine levels of IL-2 and IFN-*γ* in supernatants of Th cells incubated with quercetin were significantly reduced dose dependently but Th2 cytokines (IL-4) did not changed [90].

Despite the differences in the studied cellular model, dose of quercetin and duration of the study, all these studies demonstrated the positive effects of quercetin on cytokine production and cell-mediated immunity that suggests immunomodulatory effects of quercetin. However, more studies to evaluate the molecular mechanisms of quercetin-mediated immunomodulatory effects and its use in clinical applications are required.

*(2) In Vivo Studies.* Administration of quercetin glycosides including quercetin-3-O-*𝛃*-D-glucopyranoside (isoquercitrin, 100 mg/kg) and quercetin-3-O-rutinoside (rutin, 132 mg/kg) in immunized mice with a specific T-dependent antigen for 34 days increased proliferation of lymphocyte basal and IgM-producing lymphocytes [184]. The rutin also enhanced NK cell activity and T cells. In this study, quercetin exerted immunomodulatory influence via proliferation of B cell subsets [184].

Oliveira et al. [182] reported immunomodulatory property of quercetin in asthma induced by Blomia tropicalis. Blomia tropicalis-sensitized mice were daily treated orally with 30 mg/kg quercetin for 7 days. The number of cells, eosinophil peroxidase (EPO), IL-4, IL-5, and IL-13 in BALF were reduced in animals treated to quercetin [182]. Daily treatment of mice with oral quercitrin (20 mg/kg) during 14-day hypersensitization with OVA improved eosinophilia, IgE antibodies, and IL-5, IL-10, and TNF-*α* cytokines [185]. Therefore, immunomodulatory pretreatment with quercetin protects mice versus anaphylactic shock via downregulation of Th2-type immune responses.

Al-Rekabi et al. evaluated immunomodulatory effect of quercetin consumption (500, 1000, 1500 mg/day) in combination with azathioprine twice daily for eight weeks in active rheumatoid arthritis patients [96]. Oral use of high dose of quercetin decreased IL-6, complement protein 3 (C3) and complement protein 4 (C4) levels as well as increased IL-10 compared to lower doses of quercetin and placebo group. Intercellular adhesion molecule I (ICAM-1) was also decreased after treatment with all three doses of quercetin relative to placebo group [96]. Lack of designing of the group treated to quercetin alone and mentioning the type of randomization are some limitations of the study.

The evaluation of quercetin effect on the Th1/Th2 immune response in OVA-induced asthma in mice demonstrated that administration of quercetin for 3 days reduced the numbers of inflammatory cells in BALF [95]. In addition, MMP-9 and GATA-3 mRNA expression in lung tissues of asthmatic mice were inhibited by quercetin. Increased IFN-*γ* concentration but reduction of IL-4 and IL-5 in the BALF was also observed [95]. In a murine model of dry eye disease (DED), topical pretreatment of 0.01% quercetin, 0.1% resveratrol, and 0.01% quercetin +0.1% resveratrol decreased inflammatory response of the ocular surface, clinical symptoms, IL-1*α*, and IL-4 levels compared to DS mice treated with vehicle. In addition, quercetin reduced CD4+ T cells [186].

All the abovementioned findings reported *in vivo* studies support immunomodulatory property quercetin. However, the effects of the *P. oleracea* extract and its phytochemical components such as quercetin were studied only in a few clinical studies. Thus, further clinical studies regarding the effect of *P. oleracea* and its constituents are needed.

#### 6.2.3. Other Constituents

There are few studies about the immunomodulating effects of other constituents of *P. oleracea*. Effects of isolated three polysaccharide complexes from *P. oleracea*, silver linden, and lavender were evaluated in the *ex vivo* model of human white blood cells and the *ex vivo* murine model of Peyer's patch (PP) cells from the small intestine [187]. These complexes stimulated phagocytic leukocytes, human blood T-cell populations, and induced IL-6 production obtained from Peyer's patch cells and human white blood cells. The results demonstrated purslane polysaccharides can play a role in treatment of immune system disorders [187]. The immunomodulatory function of purslane polysaccharides (POL-P3b) was evaluated in dendritic cell (DC) vaccine for breast cancer. Treatment of tumor antigen-sensitized DC with POL-P3b (50 *µ*g/mL) increased immune responses and was effective as an immunomodulatory agent for the maturation and activation of DC [188].

Treatment of rats with ovarian cancer purslane polysaccharides demonstrated an increase of the spleen, thymocyte T, and B lymphocyte proliferation which suggest immunity-modulatory activity of this plant [60].

Assessment of effects of purslane polysaccharides on immune status in rats with N-methyl-N-nitro-N-nitrosoguanidine (MNNG) induced-gastric cancer indicated that administration of different concentrations of purslane polysaccharides (200, 400, or 800 mg/kg, as oral gavage) significantly raised proliferation of splenocytes in gastric cancer rats [189]. In addition, the serum level of cytokines, including IL-2, IL-4, and TNF-*α*, was increased after treatment with these polysaccharides. These results confirm the immunomodulatory activity of the plant that could be therapeutic value in gastric cancer [189].

Immuno-stimulating activity of a polysaccharide from of *P. oleracea* was examined on *in vivo* model by accessing the immune organ index and T lymphocyte subsets after administration of polysaccharide [190]. The findings showed an increase in immune responses through enhancement of white blood cell count, CD4+ T-lymphocytes, and CD4+/CD8+ ratios [190].

In the DSS-induced colitis mouse model, oral administration of bioactive compounds of *P. oleracea* extract (portulacanone C, cis-*n*-feruloyl-3′-methoxytyramine, and trans-*n*-feruloyltyramine) and 3% DSS led to significant reduction of cytokine levels such as TNF-*α*, IL-6, 1L-1*β*, IL-4, IL-17, and IFN-*γ* [174]. Based on these results, active compounds of *P. oleracea* have immunomodulatory properties and could be used as a new therapeutic approach in various diseases [174].

**Figure 4:**
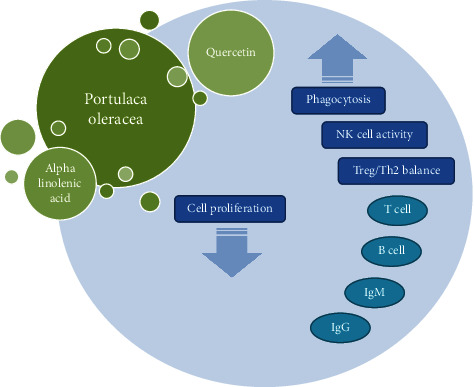
Possible mechanisms of immunomodulatory effects of *P. oleracea* and its constituents. IgM: immunoglobulin M; IgG: immunoglobulin G.

*P. oleracea* and its main constituents such as ALA, quercetin, and polysaccharide showed immunomodulatory effects by modulation of both innate (different cells from white blood cells group and NK cells) and adaptive immune (inflammatory and anti-inflammatory cytokines in the blood and tissue, B lymphocytes, T lymphocytes, antibodies formation, and Th1/Th2 balance) systems. Therefore, it can play a role in the treatment of immunological based disorders as an immune-modulatory herb.  Table 4 demonstrates immunomodulatory effects of *P. oleracea* and its components. The immunomodulatory effects of this plant and its constituents with possible mechanisms of action are also summarized in Figure 4.

## 7. Limitations and Strengths

Despite demonstrating valuable findings, there were limitations that made data interpretation difficult. The main limitation of this review article is the low number of clinical studies on anti-inflammatory, immunoregulatory, and antioxidant effects of *P. oleracea* and its constituents in various diseases. Therefore, designing further standard clinical trials with appropriate sample size can help in the better generalizability of the results. In addition, assessment of possible mechanisms related to these effects is recommended in future studies. The evaluation of the quality of the individual studies did not perform in this review, so design systematic review and meta-analysis studies can be a potential direction of research.

## 8. Conclusion

The effect of *P. oleracea* and its constituents on inflammation, oxidant/antioxidant status, and immune system was reviewed. *In vitro* and *in vivo* studies showed that the effects of this plant and its constituents such as polysaccharides and flavonoids especially quercetin could be attributed due to their anti-inflammatory, antioxidant, and immunomodulatory properties. *P. oleracea* as an antioxidant plant could be scavenged free radicals and balanced oxidant and antioxidant parameters. This herb can also inhibit inflammation and modulate the immune system via improvement of T-lymphocytes, and NK cells, and inflammatory markers (IL-4, IL-10, IFN-*γ*, and TNF-*α*) and Th1/Th2 balance. Reviewed studies suggest the potential therapeutic value of *P. oleracea* for treatment of disorders related to inflammation, oxidant/antioxidant status, and immune system imbalance.

## Figures and Tables

**Table 1:** Compounds isolated from *P. oleracea*.

Chemical class	Chemical compound	Parts of plants	References
Flavonoids	Portulacanones A–D	Aerial parts	[24]
	Kaempferol	Whole plant	[25]
	Apigenin	Whole plant	[25]
	Luteolin	Whole plant	[25]
	Myricetin	Whole plant	[25]
	Quercetin	Whole plant	[25]
	Genistein	Whole plant	[26]
	Genistin	Whole plant	[26]

Alkaloids	Oleracein A–E	Whole plant	[27]
	Oleorecins I and II	Stems	[27]
	(3R)-3,5-Bis(3-methoxy-4-hydroxyphenyl)-2,3-dihydro-2(1 H)-pyridinone	Aerial parts	[10]
	1,5-Dimethyl-6-phenyl-1,2-dihydro-1,2,4-triazin-3(2H)-one	Aerial parts	[10]
	Trollisine	Aerial parts	[28]
	Adenosine	Whole plant	[27]
	Aurantiamide acetate	Aerial parts	[28]
	Aurantiamide	Aerial parts	[28]
	Scopoletin	Aerial parts	[29]
	Dopamine	Stem, leaf, and seed	[30]
	Noradrenalin	Stem, leaf, and seed	[31]
	Oleracone	Whole plant	[4]

Terpenoids	Portuloside A and B	Aerial parts	[32, 33]
	Portulene	Aerial parts	[34]
	Lupeol	Aerial parts	[34]
	(3S)-3-O-(*β*-D-Glucopyranosyl)-3,7-dimethylocta-1,6-dien-3-ol	Aerial parts	[33]
	(3S)-3-O-(*β*-D-Glucopyranosyl)-3,7-dimethylocta-1,5-dien-3,7-diol	Aerial parts	[33]
	(2*α*,3*α*)-3-{[4-O-(*β*-D-Glucopyranosyl)-*β*-D-xylopyranosyl]oxy}-2,23-dihydroxy-30-methoxy-30-oxoolean-12-en-28-oic acid	Aerial parts	[35]
	(2*α*,3*α*)-2,23,30-Trihydroxy-3-[(*β*-D-xylopyranosyl)oxy]olean-12-en-28-oic acid	Aerial parts	[35]
	Friedelane	Aerial parts	[35]

Carotenoids	*α*-Carotene	Leaf	[36]
	*β*-Carotene	Leaf	[37]
	Lutein	Leaf	[36]
	Zeaxanthin	Leaf	[36]

Fatty acids	*α*-Linolenic acid	Leaf	[38]
	Linolenic acid	Leaf	[39]
	Palmitic acid	Leaf	[37]
	Stearic acid	Leaf	[37]
	Oleic acid	Leaf	[37]
	p-Coumaric acid	Whole plant	[27]
	Ferulic acid	Whole plant	[27]
	Docosapentaenoic acid	Stem	[39]
	Eicosapentaenoic acid		[40]
	Docosahexaenoic acid		[40]
	Catechol		[40]
	Caffeic acid	Aerial parts	[41]
	Oxalic acid	Leaf	[42]
	Lonchocarpic acid		[29]

Vitamins	Vitamin A	Leaf	[39]
	B-complex	Leaf	[39]
	Vitamin C	Leaf	[39]
	*α*-Tocopherol	Leaf	[37]
	Hesperidin	Leaf	[43]

Minerals	Potassium	Stem and leaf	[44]
	Magnesium	Leaf	[39]
	Calcium	Root, stem, and leaf	[44]
	Manganese	Root, stem, and leaf	[44]
	Phosphorus	Root, stem, and leaf	[44]
	Copper	Root, stem, and leaf	[39]
	Selenium	Leaf	[39]
	Zinc	Leaf	[44]
	Iron	Root, stem, and leaf	[44]

Carbohydrates	Polysaccharides	Aerial parts	[45]

Glycosphingolipids	Portulacerebroside A	Aerial parts	[46]

Enzymes	Glutathione	Leaf	[37]

Pigments	Chlorophyll	Root and leaf	[40]

Hormones	Melatonin	Leaf	[38]

**Table 2:** Anti-inflammatory effects of *P. oleracea* and its constituents.

Extracts	Effective doses	Model of study	Effects	References
Hydroethanolic	160 *µ*g/ml	Lymphocyte	↓ IL-4, IL-10, and NO production	[54]
Aqueous	100 *µ*g/ml	Vascular endothelial cells	↓ mRNA expressions of MCP-1 and IL-8	[55]
Ethanol	200 *µ*g/ml	RAW 264.7 cells	↓ TNF-*α*, IL-1*β*, and IL-6	[98]
POL-P3b	250 *µ*g/ml	DCs	↑ IL-12, TNF-*α*, and IL-10	[57]
Ethanol	0.5 and 1 mg/ml	RAW 264.7 cells	↓ NO production and mRNA expression of TNF-*α* and IL-1-*β*	[58]
Aqueous	1–200 *μ*g/ml	HUVECs	↓ TNF-*α*-induced overexpression of ICAM-1, VCAM-1, and E-selectin	[55]
Hydroalcoholic	100 *μ*g/ml	PBMCs	↓ TNF-*α* and IL-6	[59]
Polysaccharide	600 *µ*g/ml	Rat	↑ T lymphocytes and B lymphocytes	[60]
Hydroethanolic	1, 2, and 4 mg/ml	Rat	↓ TP, PLA2, and IgE	[62]
Hydroethanolic	1, 2, and 4 mg/ml	Rat	↓ Serum levels of NO_2_, NO_3_, and total WBC count	[63]
Hydroalcoholic	50, 100, and 200 mg/kg	Rat	↓ IL-1*β*, IL-6, TNF-*α*, PGE2, and TGF-*β*	[64]
			↑ IL-10	
Aqueous	300 mg/kg/day	Mice	↓ NF-*κ*B p65 activation	[65]
			↑ Expression of TGF-*β*1 and ICAM-1	
Aqueous	300 mg/kg/day	Mice	↓ Overexpression of VCAM-1, ICAM-1, E-selectin, MMP-2, and ET-1	[66]
Hydroalcoholic	100 and 200 mg/kg	Rat	↓ Pain-related behaviors and contents of TNF-*α* and IL1*β*	[68]
Hydroalcoholic	400 mg/kg	Rat	↓ Level of TNF-*α*	[69]
			↑ Improved memory	
Methanolic	500 mg/kg	Rat	↓ TNF-*α* and TGF-*β*	[70]
Aqueous	1, 2, and 4 g/kg	Mice	↓ TNF-*α*, IL-1b, and IL-6	[71]
Aqueous	400 mg/kg/day	Mice	↓ TNF-*α*, IL1*β*, and NF-*κ*B	[72]
Purslane powder	8% purslane + 100 mg/l CdCl_2_ in water	Mice	↓ TNF-*α*, IL-6, IL-1*β*, and IFN-*γ*	[73]
Hydroethanolic	100 and 300 mg/kg/day	Rat	Improved MDA, TNF-*α*, and TGF-*β*1	[74]
Aqueous and ultrasound-assisted ethanol	3 mL 1 g/mL	Mice	Inhibited TNF-*α*, IFN-*γ* and IL-4	[75]
Hydroethanolic	100 and 300 mg/kg/day	Rat	↓ IL-6, TNF-*α*, and IL-1*β*	[76]
			↑ IL-10	
ALA	125 *μ*M	HCE cells	↓ Protein and mRNA levels of IL-6, IL-8, IL-1*β*, and TNF-*α*	[80]
ALA	100 *μ*M	THP-1 cells	↓ Secretion and mRNA levels of IL-6, IL-1*β*, and TNF*α*. ALA	[81]
ALA	5 and 10 mg/kg	RAW 264.7 cells	↓ Production of NO. and inhibited iNOS, COX-2, and TNF-*α* gene expressions induced by LPS	[82]
ALA	6.5% of energy	PBMCs of patients	↓ Production of IL-6, IL-1*β*, and TNF-*α*	[83]
ALA	60 mg/kg	Mice	Suppressed NF-*κ*B and IL-1*β*	[84]
ALA	10% ALA	Mice	↓ IL-1*β*, IL-6, and TNF*α*	[85]
ALA	500 and 2000 mg/kg	Mice	↓ IL-6 and IL-1*β* in nasal mucosa	[86]
ALA	8 g	Human	↓ C-reactive protein (CRP), serum amyloid A (SAA), and IL-6 levels	[87]
Quercetin	100 *µ*g/ml	DCs	↓ IL-1*α*, IL-1 *β*, IL-6, IL-10, IL-12 p70), and chemokines (MCP-1, MIP-1 a, and MIP-1 b)	[89]
Quercetin	40 *µ*M	Th cells	↓ IL-2, IFN-*γ*, and IL-2Ra expressions	[90]
Quercetin	10, 25 and 50 *µ*M	PBMC	↓ IL-4	[91]
			↑ IFN-*γ*	
Quercetin	50 *µ*M	PBMC	↓ IL-1*β*, TNF-*α*, MMP-9, and TIMP-1	[92]
Quercetin	50 *µ*M	BMDM	↓ iNOS expression, TNF-*α*, IL-1*β* protein expression, and *I*_*k*_B-*α* phosphorylation	[94]
Quercetin	10–30 *μ*mol/L	HUVEC	↓ NF*κ*B and expression of VCAM-1 and E-selectin	[93]
Quercetin	0.1%, w/w	Mice	↓ IL-1R, CCL8, IKK, serum amyloid A, and fibrinogen	[93]
Quercetin	1 mg/kg/	Rat	↓ TNF-*α*, IL-1*β* expression, and iNOS	[94]
Quercetin	1 mg/kg	Mice	↓ IL-4, IL-5 secretion, mRNA expression of MMP-9, and EPO	[95]
			↑ IFN-*γ*	
Quercetin	1500 mg/day, p.o.	Human	↓ IL-6 and ICAM-1	[96]
			↑ IL-10	
Quercetin	50 mg/kg	Mice	↓ Th17 cells and gut inflammation	[97]
			↑ Treg cells	

DCs: dendritic cells; HUVECs: human umbilical vein endothelial cells; PBMCs: human peripheral blood mononuclear cells; IL: interleukin; NO: nitric oxide; MCP: monocyte chemoattractant protein; TIMP-1: tissue inhibitor of metalloproteinases-1; CCL8: chemokine (C-C motif) ligand 8; IKK: I*κ*B kinase; TNF-*α*: tumor necrosis factor-alpha; NF-*κ*B: Nuclear factor-kappa B; TP: total protein; PLA2: phospholipase A2; PGE2: prostaglandin E2; TGF-*β*: transforming growth factor beta; MMP: matrix metalloproteinase; ET-1: endothelin-1; EPO: erythropoietin; ICAM-1: intercellular adhesion molecule-1; VCAM-1: vascular cell adhesion molecule-1; HCE: human corneal epithelial; ALA: alpha-linolenic acid.

**Table 3:** Antioxidant effects of *P. oleracea* and its constituents.

Extracts/constituents	Doses	Model of study	Effects	References
Aqueous, methanolic, and ethanolic extracts		*In vitro*	**↑** DPPH scavenging capacity and FRAP	[115]
Aqueous extracts of leaves, stems, and flowers		*In vitro*	↑ DPPH scavenging capacity	[116]
Aqueous and ethanolic extracts	100–400 *µ*g/ml	Free radical-induced hemolysis of RBCs in rats	↓ Rate of AAPH-induced hemolysis	[127]
	1200, 1800 *µ*g/ml		↑ Lag time of AAPH-induced hemolysis	
			↓ RBC damages	
Ethanolic extract of leaves		*In vitro*	↑ ABTS and DPPH scavenging capacity	[117]
Methanolic extract		*In vitro*	↑ DPPH scavenging capacity	[118]
Methanolic extract		*In vitro*	↑ DPPH scavenging capacity	[119]
			↓ Lipid peroxidation	
Methanolic extract of leaves		*In vitro*	↑ DPPH scavenging capacity	[120]
			Inhibition ratio of the linoleic acid oxidation	
Methanolic extract		*In vitro*	↑ Electron donating ability and SOD-like ability	[121]
Fresh and dried hydroalcoholic extracts		*In vitro*	↑ ABTS and DPPH scavenging capacity	[122]
Fresh and dried leaves		*In vitro*	↑ ABTS and DPPH scavenging capacity	[123]
Five fractions obtained from the crude extract		*In vitro*	↑ Trolox equivalent antioxidant capacity (TEAC)	[124]
			↓ Lipid peroxidation	
Aqueous juice	1.5 ml/kg, orally for 12 days	Normal rats	↓ Hepatic and renal MDA	[114]
			↓ Testicular nitrite/nitrate	
			↑ Hepatic and testicular GSH	
			↑ Hepatic, renal, and testicular SOD and CAT	
Aqueous extract	100 and 200 mg/kg, intragastrically	High-fat diet-induced mice	↓ Blood TBARS	[125]
			↑ Blood GSH	
			↑ Blood and liver SOD, CAT, and GPx	
			↓ Liver ALT and AST	
Aqueous extract	300 mg/kg, intragastrically for 5 days	Renal ischemia reperfusion injury (IRI) in rats	↓ ALT, ALP, and LDH	[126]
Ethanolic extract	0.01, 0.05, 0.1, and 0.15 g/kg, intragastrically for 30 days	Carbon tetrachloride (CCl4)-induced hepatic toxicity in rats	↓ ALT, AST, ALP, and GGT	[128]
			↑ SOD	
Ethanolic extract of leaves	25, 50, and 100 mg/kg, orally	Alcoholic liver disease rat's model	↓ AST, ALT, ALP, and GGT	[117]
			↓ TBARS and lipid hydroperoxides	
			↑ Vitamin C, vitamin E, and GSH	
			↑ SOD, CAT, GPx, and GST	
Ethanolic extract	4 mg/kg, orally	Neurotoxicity induced by MeHg in cerebellum and cortex of rats	↓ TBARS	[129]
			↑ GSH, GPx, CAT, and SOD	
Fresh and dried leaves	200 and 400 mg/kg, orally for 3 weeks	STZ-induced C57BL/6J diabetic mice	↓ MDA	[130]
			↑ SOD	
Fresh juice	300 mg/kg, orally	Paracetamol-induced hepatic toxicity in rats	↓ Hepatic TBARS content	[131]
			↑ GSH, CAT, and SOD	
Hydroethanolic extract	1, 2, and 4 mg/mL	Asthmatic rat model	↓ MDA	[132]
			↑ SOD, CAT, and thiol	
Purslane ethanolic extract and chicory water extract	100 mg/kg for each	Glucocorticoid-induced testicular and autophagy dysfunction in rats	↓ MDA	[133]
			↑ GSH, GST, and GPx	
Seed extract	200 and 400 mg/kg	Acrylamide-induced testicular toxicity in rats	↑ SOD and GSH	[134]
			↓ MDA	
Hydroalcoholic extract	25, 50, and 100 mg/L	*In vitro*	↓ Intracellular ROS	[135]
			↑ Motility of sperm	
Hydroalcoholic extract	400 mg/kg	Thyrotoxic rat model	↑ Thiol, SOD, and CAT	[136]
			↓ MDA	
Plant sterol ester of *α*-linolenic acid (PS-ALA)	0.1 mM PS-ALA	HepG2 cells induced by oleic acid	↓ ROS production	[137]
ALA	1, 10, 33, 49, and 64%, orally for 21 days	Rats fed sunflower, canola, rosa mosqueta, and sacha inchi oils	**↑** GSH and GSSG, hepatic and plasma content of protein carbonyls, F-2 isoprostanes, TBARS, SOD, CAT, GPx, and GR	[138]
ALA	150 *μ*g/kg	Amyloid-beta peptide-induced oxidative stress in rats	↓ MDA and NO	[139]
			↑ CAT activity and glutathione content in hippocampus	
Quercetin		Iron-loaded hepatocyte cultures *in vitro*	↓ MDA and LDH	[140]
Quercetin	2.5, 5 and 7.5 *µ*M	Copper-catalysed human LDL oxidation *in vitro*	↓ LDL oxidation	[141]
Quercetin		Human erythrocytes *in vitro*	↓ lipid peroxidation, hemolysis, and GSH	[142]
Quercetin	50 or 100 *µ*M	Glucose oxidase-mediated apoptosis	↓ NF-kappaB, AP-1, and p53	[143]
Quercetin	10, 20, 50, 100, or 1000 *µ*M	*In vitro*	↓ Hydroperoxides	[144]
Quercetin		*In vitro*	**↑** FRAP and ABTS	[145]
Quercetin-loaded nanoparticles		*In vitro*	**↑**Anti-superoxide formation and DPPH scavenging capacity	[146]
			↓ Superoxide anion	
Quercetin-loaded nanoparticles		*In vitro*	**↑** DPPH scavenging capacity	[147]
Quercetin	2%, orally for 21 days	Rats adapted to a semipurified diet supplemented with quercetin	**↑** ABTS	[148]
Quercetin	15 mg/kg, i.p. for 4 weeks	STZ-induced diabetic rats	↓ MDA and NO	[149]
			**↑** SOD, CAT, and GPx	
Quercetin	75 mg/kg, i.p. for 10 days	Cyclophosphamide-induced hepatotoxicity in rats	↓ MDA and PCO	[150]
Quercetin	50 mg/kg, for 10 days	Bile duct ligation-induced liver injury in rats	↑ Glutathione peroxidase	[151]
			↓ Oxidation of proteins	
Quercetin	50 mg/kg, intragastrically	Normal rats	↓ Plasma antioxidant status	[152]
Quercetin	10 mg/kg, i.p. for 14 days	STZ-induced diabetic rats	**↑** Brain GSH, hepatic GPx, hepatic lipid peroxidation, renal and cardiac GPx, and cardiac CAT	[153]
			↓ Hepatic GSH	
Quercetin	50 mg/kg, i.p.	Ethanol-induced gastric lesions in rats	↓ MDA	[154]
			**↑** SOD, CAT, and GPx	
Quercetin	100 mg/kg, for 14 days	Rat model of tramadol intoxication	Improved MDA, SOD, NOx in the heart, liver, adrenal, and kidney	[155]
Quercetin	50 mg/kg, orally	Radiation-induced hepatotoxicity and renal toxicity in rats	↓ MDA level in the liver and kidney	[156]
Quercetin	10 mg/kg, gavage	Mice exposed to cigarette smoke	↓ Inflammatory cytokines	[157]
			**↑** SOD and CAT	
			↓ Myeloperoxidase	
Quercetin	25 mg/kg, gavage, for 40 days	Dental pulp of the streptozotocin-diabetic rats	Improved CAT, SOD1, GPX1, and TAC levels	[158]
Seed oil	3–20 mg/ml	*In vitro*	↑ Hydroxyl free radical and DPPH scavenging capacity	[159]
Some component of leaves		*In vitro*	↑ DPPH scavenging capacity	[160]
Polysaccharide fractions; POP II and POP III		*In vitro*	↑ Antioxidant activities in cell-free radical generating systems and cell-mediated radical generating systems	[161]
Polysaccharide fraction	25 and 50 mg/kg, orally	STZ-induced diabetes in rats	↓ TBARS	[162]
			↑ GSH, GPx, CAT, and SOD	
Phenolic alkaloids: oleracein A (OA), oleracein B (OB) and oleracein E (OE)		Hydrogen peroxide-induced lipid peroxidation in rat brain	↑ DPPH scavenging capacity	[41]
			↓ MDA	
Diet supplemented with leaves	240 g/kg of leaves, orally	Oxidative stress induced by vitamin A deficiency in rats	↑ DPPH scavenging capacity	[163]
			↑ GSH and GSSG	

ABTS: antioxidant capacity determined by radical cation; FRAP: ferric-reducing antioxidant power; TBARS: thiobarbituric acid reactive substances; PCO: protein carbonyl; DPPH: 1,1-diphenyl-2-picryl-hydrazyl; CAT: catalase, SOD: superoxide dismutase, MDA: malondialdehyde; GSH: glutathione; GPx: glutathione peroxidase; GR: glutathione reductase; ALT: alanine aminotransferase; AST: aspartate aminotransferase; ALP: alkaline phosphatase; LDH: lactate dehydrogenase; AAPH: 2, 2′ azobis (2-amidinopropane) hydrochloride; GGT: gamma-glutamyl transferase; GSSG: glutathione disulfide; STZ: streptozotocin; ALA: alpha-linolenic acid.

**Table 4:** Immunomodulatory effect of *P. oleracea* and its constituents.

Extracts/constituents	Doses	Model of study	Effects	References
Hydroethanolic extract of *P. oleracea*	160, 40, and 10 *µ*g/ml	Nonstimulated and PHA-stimulated human lymphocytes	↓ Percentage of cell proliferation and serum levels of IL-4, IL10, and IFN-*γ*	[54]
			↑ IFN-*γ*/IL-4 and Treg/Th2 (IL-10/IL-4) balances	
Ethyl acetate and chloroform extracts of *P. werdermannii* and *P. hirsutissima*	1, 10, and 100 *μ*g/ml	Concanavalin A-induced BALB/c-isolated splenocytes	↓ Cell proliferation	[168]
Ethyl acetate extract of *P. oleracea*	5 and 50 mg/kg	Mice model of cyclophosphamide-induced immune-suppression	↑ Phagocytosis	[169]
			↑ Proliferative response in splenic lymphocytes	
Extract of *P. oleracea*	1, 2, and 4 mg/ml	Rat model of asthma	↑ INF-*γ*/IL4 ratio	[170]
Hydroethanolic extract of *P. oleracea*	1, 2, and 4 mg/ml, in drinking water	Rat model of asthma	↓ BALF levels of IgE	[62]
Purslane/pumpkin seed mixture	Ratio of 5/1 of pumpkin and purslane	Hypercholesterolemic rats	↑ Levels of IgG and IgM	[171]
Ethanolic extract	200 mg/kg	Mice model of acetic acid-induced ulcerative colitis	↓ IL-1, IL-6, IL-17, TNF-*α*, IFN-*γ*, NF-*κ*B, and myeloperoxidase activity	[172]
*P. oleracea* and *Perilla frutescens* seed complex extracts	0, 5, 10, 30, 50, 100, and 300 *μ*g/ml	Cyclophosphamide-stimulated rat and isolated spleen cells	↑ Immune cells and splenic recovery	[173]
			↑ Proliferation of splenocyte and inflammatory cytokines (IL-2, IL-12, TNF-*α*, and IFN-*γ*), and NK cell activity	
Ethyl acetate and ethanol extracts of *P. oleracea*	30 or 60 *μ*g/ml (*in vitro*)	LPS-induced inflammatory responses in macrophages	↓ Serum levels of TNF-*α*, IL-6, and 1L-1*β*	[174]
	100, 300, and 500 mg/kg/day (*in vivo*)	Mice model of colitis induced by DSS		
*P. oleracea*	20 mg/ml	2,4-dinitrofluorobenzene induced-atopic dermatitis mice model	↓ Serum levels of histamine HIS and Ig E	[175]
			↓ TNF-*α*, IL-4, and IFN-*γ*	
			↓ Mast cell infiltration	
ALA	0.7, 10.6, and 37% dietary intake, for 4 weeks	Fatty acids-rich diet in mice model	↑ IFN-*γ*/IL-4 and Th1/Th2 balances	[176]
ALA	0, 100, 200, 300, and 400 g/d infused to the duodenum	Dairy cows infused to ALA	↑ Serum IgG and IFN-*γ*	[11]
			↓ IL-4 and PGE2	
ALA	178 g/kg, dietary intake, for 6 weeks	High-fat diet in rat model	Modifying the spleen lymphocyte functions	[177]
ALA	178 g/kg lipid contained 4.4 g ALA, dietary intake	High-fat diet in rat model	Modifying cell-mediated immune response	[178]
ALA	0.2 and 0.4 mg/ml, in drinking water	Sensitized rats with ovalbumin	↑ IFN-*γ* and IFN-*γ*/IL-4	[179]
			↓ IL-4	
Quercetin	20, 40, and 80 *μ*M	Human glomerular mesangial cells (HGMCs)	↓ Expression of nuclear factor-*κ*B p65 and IKK*β*	[180]
			↑ Expression of I*κ*B*α* inhibited the expression of PTX3 antibody	
Quercetin-3-O-*α*-L-rhamnopyranoside	150 *μ*g/ml, for more 48 h	Propagated influenza virus in Madin–Darby canine kidney (MDCK) cells	↓ Expression of TNF-*α*	[181]
			↑ Expression of IL-27	
Quercetin	3.5, 7.5, and 15 *μ*g/ml	Murine model of Blomia tropicalis-induced asthma	↓ IL-4, IL-5, and IL-13 cytokines in supernatants of cells	[182]
Shikimic acid and quercetin combination	10 and 100 nM, for 24 h	Peripheral blood mononuclear cells	↑ IL-8 and IL-6 cytokine levels	[183]
Quercetin	6.25, 12.5, 50, and 100 *µ*g/ml, for 24 h	DCs simulated by LPS	↓ IL-1a, IL-1b, IL-6, IL-10, and IL-12 p70	[89]
			↓ MCP-1, MIP-1a, MIP-1b, and RANTES	
			↓ CD40, CD80, and CD86	
Quercetin	1, 10, and 50 *µ*M, for 24 h	LPS-stimulated bone marrow-derived macrophages	Inhibited expression of TNF-*α* and IL-1*β* proteins, IkB-*α* phosphorylation and iNOS expression	[94]
Quercetin	0.5–50 *µ*M, for 24–72 h	Blood mononuclear cells from normal subjects	↑ IFN-*γ*	[91]
			↓ IL-4 markedly	
Quercetin	5–200 *µ*M, for 48 h	PBMC isolated from multiple sclerosis patients	↓ PBMC proliferation	[92]
			↓ IL-1*β* and TNF-*α*	
			↓ MMP-9/TIMP-1 ratios	
Quercetin	40 *µ*M, for 24 h	T helper cells	↓ IL-2 and IFN-*γ*	[90]
Quercetin-3-O-𝛃-D-glucopyranoside (isoquercitrin) and quercetin-3-O-rutinoside (rutin)	Isoquercitrin (100 mg/kg)	Immunized mice and treated with isoquercitrin and rutin	↑ Lymphocyte basal proliferation	[184]
	Rutin (132 mg/kg), dietary intake, for 34 days		↑ The number of IgM-producing lymphocytes	
			↑ NK activity and T cells	
Quercetin	30 mg/kg, orally for 7 days	Blomia tropicalis-sensitized mice	↓ Number of cells, EPO and IL-4, IL-5, and IL-13 cytokines	[182]
Quercetin	20 mg/kg, intragastric gavage, for 14 day	Hypersensitization with ovalbumin in mouse model	↓ IgE antibodies	[185]
			↓ Eosinophilia	
			Impaired production of the IL-5, IL-10, and TNF-*α* cytokines	
Quercetin	500, 1000, and 1500 mg/day, orally, twice daily for 8 weeks	Active rheumatoid arthritis patients	↓ IL-6, C3, C4, and ICAM-1	[96]
			↑ IL-10	
Quercetin	8 and 16 mg/kg/day, i.p., for 3 days	OVA-induced asthma model mice	↓ Inflammatory cells	[95]
			↓ MMP-9 and GATA-3 mRNA	
			↓ IL-4 and IL-5	
			↑ IFN-*γ*	
Quercetin	0.01%	Murine model of dry eye disease (DED)	↓ Inflammatory response	[186]
			↓ Clinical symptoms	
			↓ IL-1*α* and IL-4 levels	
			↓ CD4+ T cells	
Polysaccharide complexes from purslane, silver linden, and lavender	10 and 100 *µ*g/ml	*Ex vivo* model of human white blood cells	↑ Human blood T-cell populations (CD4+/CD25+ and CD8+/CD25+), phagocytic leukocytes (CD14+ and CD64+ cells)	[187]
		*Ex vivo* murine model of Peyer's patch (PP) cells from the small intestine	↑ IL-6 production from human white blood cells and Peyer's patch cells	
Polysaccharide of *P. oleracea* (POL-P3b)	50 *µ*g·mL^−1^	Tumor antigen-sensitized dendritic cells	↑ Immune responses	[188]
Polysaccharide of *P. oleracea*	0.3,0.6, 0.9, and 1.2 mg/ml	Rat model of ovarian cancer	↑ Spleen, thymocyte T and B lymphocyte proliferation	[60]
Polysaccharide of *P. oleracea*	200, 400, or 800 mg/kg, oral gavage, once daily for 15 weeks	N-methyl-N-nitro-N-nitrosoguanidine (MNNG)-induced gastric cancer rats	↑ Splenocytes proliferation	[189]
			↑ Serum level of IL-2, IL-4 and TNF-*α*	
Polysaccharide of *P. oleracea*	25, 50, and 100 mg/kg, intragastrically, once daily for 10 days	Mice model transplanted with sarcoma 180	↑ White blood cell count, CD4+ T-lymphocytes, and CD4+/CD8+ ratio	[190]
Portulacanone C, trans-*n* feruloyltyramine, cis-*n*-feruloyl-3′-methoxytyramine	3 mg/kg, orally, for 5 days	Mice model of DSS-induced colitis	↓ Levels of TNF-*α*, IL-6, 1L-1*β*, IL-4, IL-17, and IFN-*γ*	[174]

PHA: phytohemagglutinin; DSS: dextran sulphate sodium; LPS: lipopolysaccharide; IL: interleukin; INF-*γ*: interferon gamma; Th: T helper cells; BALF: bronchoalveolar lavage fluid; IgE: immunoglobulin E; IgM: immunoglobulin M; IgG: immunoglobulin G; TNF-*α*: tumor necrosis factor-alpha, NF-*κ*B: nuclear factor-kappa B; NK: natural killer cell; EPO: eosinophil peroxidase; C3: complement protein 3; C4: complement protein 4; ICAM-1: intercellular adhesion molecule I; LPS: lipopolysaccharides; DCs: dendritic cells; i.p.: intraperitoneally; PBMC: peripheral blood mononuclear cells; ALA: alpha-linolenic acid.

## Data Availability

No data were used to support the findings of this study.
